# Highly Porous Holey Carbon for High Areal Energy Density Solid-State Supercapacitor Application

**DOI:** 10.3390/mi13060916

**Published:** 2022-06-09

**Authors:** Christine Young, Hong-Ting Chen, Sahn-Ze Guo

**Affiliations:** Functional Nanoporous Materials Laboratory, Department of Chemical and Materials Engineering, National Yunlin University of Science and Technology, Yunlin 640, Taiwan; m10915048@yuntech.edu.tw (H.-T.C.); b10715126@yuntech.edu.tw (S.-Z.G.)

**Keywords:** biomass, energy conversion, carbon, supercapacitor, all solid-state supercapacitor

## Abstract

Biomass materials are perceived as sustainable, carbon-rich precursors for the fabrication of carbon materials. In this study, we demonstrated the capacitance performance of biomass-derived carbon, produced by using golden shower tree seeds (GTs) as carbon precursors and potassium ferrate (K_2_FeO_4_) as the activation agent. The as-prepared porous carbon (GTPC) possessed an ultrahigh specific surface area (1915 m^2^ g^−1^) and abundant pores. They also exhibited superior electrochemical performance, owing to their well-constructed porous structure, high surface area, and optimized porous structure. Optimized activated carbon (GTPC-1) was used to assemble a symmetric solid-state supercapacitor device with poly(vinyl alcohol) (PVA)/H_2_SO_4_ as a solid-state gel electrolyte. The device exhibited a maximum areal energy density of 42.93 µWh cm^−2^ at a power density of 520 µW cm^−2^.

## 1. Introduction

Owing to shortages of fossil fuels and the increasing issue of environmental pollution, sustainable energy research is becoming important for future clean energy systems. Renewable energies such as wind power and solar power are intermittent due to their fluctuating natures. Therefore, energy storage devices are required to mitigate the imbalance between energy production and demand. Moreover, with the rapid advances in miniaturized and wearable electronics, the design of compatible energy storage devices has become more vital than ever [1]. Supercapacitors, as promising energy storage devices, have advantageous characteristics, such as a fast charge/discharge rate, a long cycle life, and the provision of relatively higher power densities than batteries. Owing to the growing demand for portable electronic devices, all-solid-state flexible supercapacitors are emerging as the most practical and feasible technologies for such devices [2]. Currently, the electrode materials for supercapacitors mainly consist of carbonaceous materials. In particular, carbon nanotubes and graphene are widely used, as they exhibit unique architectures, high electrical conductivities, and exceptional physicochemical properties [3,4,5]. However, their preparation processes usually require several complicated steps, meticulous control, and expensive equipment [6]. For example, high-quality graphene is usually fabricated using chemical vapor deposition techniques, which are expensive and produce very low yields. In addition, the common precursors are methane, acetylene, and ethylene, which are extracted from fossil fuels [6]. Hence, the development of green, large-scale, and low-cost preparation methods for carbon materials is important for establishing reliable and environmentally benign supercapacitors.

Compared with artificial carbon materials, biomass-derived carbon materials have several advantages. They are not only bountiful, inexpensive, environmentally friendly, and suitable for large-scale production, but they are also highly porous, making them good for electrochemical applications. For example, Jiang et al. developed porous carbon fibers from disposable bamboo chopsticks with a specific surface area of 808.25 m^2^ g^−1^ via hydrothermal, activation, and carbonization treatments [7]. The carbon fibers exhibited good performance as electrodes in Li-ion batteries. Duan et al. prepared nanofibrous microspheres with a high specific surface area (over 1000 m^2^ g^−1^) using chitin derived from discarded seafood (crab and shrimp shells), and then applied them in supercapacitor applications [8]. Zhang synthesized porous graphitic carbon sheets grafted on cellulose fibers, providing a large specific surface area (1515.6 m^2^ g^−1^) and high specific capacitance [9]. These results revealed that biomass-derived carbon materials can achieve ultrahigh porosities, and are candidates for electrochemical energy storage applications.

The porosity of carbon materials is a dominant factor controlling their electrochemical performance. Generally, carbon materials can be classified into three categories according to their pore size: macroporous (>50 nm), mesoporous (2–50 nm), and microporous (<2 nm). The presence of micropores can significantly increase the specific area and porosity, thereby enhancing the specific capacitance of high-performance electrochemical supercapacitors [10]. However, in some cases, the channels in the microporous materials are so narrow that the electrolyte guest species cannot access the interior pores, leading to a low contribution to the double-layer capacitance. Consequently, the development of mesopores and macropores is crucial, as their presence can efficiently improve ion transportation, accommodate larger ions, and minimize the diffusion distance between the electrolyte and electrode.

Chemical activation is a common approach for obtaining porous structures for introducing additional porosity into carbons [11]. Many activation agents are helpful for enlarging and expanding the porosity, such as KOH, NaOH, ZnCl_2_, and steam [12,13,14,15]. Among these, potassium ferrate (K_2_FeO_4_) is favorable for the fabrication of graphitized porous carbon [16]. During co-pyrolysis, K_2_FeO_4_ decomposes into KOH and zero-valent Fe. At high temperatures, KOH can be used etch carbon atoms, generating a large number of pores and contributing to a large specific surface area [17]. In contrast, Fe can act as a graphitization catalyst to promote the conversion of amorphous carbon into semi-graphitized and graphitized carbon [18]. This type of structural conversion leads to an increase in the degree of graphitization and enhanced electrical conductivity. Therefore, it is practical to use K_2_FeO_4_ to fabricate biomass-derived carbons with hierarchically porous structures.

The golden shower tree (*Cassia fistula*) is a flowering plant that is abundant on the National Yunlin University of Science and Technology campus in Taiwan. In this study, we fabricated activated carbon with high porosity from golden shower tree seeds (GTs). First, the GTs were ground with K_2_FeO_4_ into a powder. The mixture was then carbonized at 800 °C under a nitrogen atmosphere. Consequently, the biomass-derived carbon showed a holey morphology, and exhibited the highest specific area of 1915 m^2^ g^−1^. When evaluated as electrode materials for supercapacitors, this green, activated carbon showed a high specific capacitance (156.9 F g^−1^ at a scan rate of 5 mV s^−1^). An all-solid-state supercapacitor cell was fabricated using poly(vinyl alcohol) (PVA)/H_2_SO_4_ as the solid-state gel electrolyte, and the optimized biomass-derived carbon as the electrode material. The cell exhibited a high energy density, with a maximum areal energy density of 42.93 µWh cm^−2^ at a power density of 520 µW cm^−2^, and excellent electrochemical stability (~90.08%) after 1000 cycles at a high current density of 1 A g^−1^. Considering their electrochemical performance, ease of large-scale manufacturing, and environmental friendliness, this method offers a promising approach for fabricating porous, biomass-derived carbon electrodes for lightweight and low-cost energy storage devices.

## 2. Experimental Section

### 2.1. Chemicals

Potassium ferrate (K_2_FeO_4_; >90 wt %) and 1-methyl-2-pyrrolidinone were purchased from Sigma-Aldric (St. Louis, MO, USA). Hydrofluoric acid (HF; >48 wt %) was obtained from Honeywell Fluka (Charlotte, NC, USA). Potassium hydroxide (KOH) was purchased from Duksan Reagents (Ansan, Kyeonggi, Korea). Super-P was obtained from Alfa Aesar (Haverhill, MA, USA). Carbon cloth (WOS1009) was purchased from CeTech Co. Ltd (Taichung City, Taiwan).

### 2.2. Synthesis of Biomass Derived Carbon

The natural GTs used in this study were collected from the local campus at the National Yunlin University of Science and Technology. The GTs were ground into powder and washed with deionized (DI) water and ethanol several times to remove impurities. The samples were collected after drying at 70 °C overnight. Then, the GTs and K_2_FeO_4_ were ground in a mortar at weight ratios of 1:1 and 1:2, respectively, to mix them completely. The mixed samples were subsequently carbonized in a tube furnace at 800 °C for 2 h at a heating rate of 5 °C min^−1^ under a nitrogen atmosphere. The resultant product was washed with an 8 wt % HF aqueous solution to remove the remaining metal species. Finally, the prepared golden shower tree derived porous carbon (GTPC) was purified with DI water several times until the filtrate became neutral, and was then dried at 70 °C overnight. The resultant samples were denoted as GTPC-1 and GTPC-2, according to the weight ratio of the GTs to K_2_FeO_4_. For comparison, the activation agent was changed to KOH at a weight ratio of 1:1, and the synthesis method was the same as above. The resultant product was denoted as GTPC-KOH. The GTs were also directly carbonized without any activation treatment, and the corresponding obtained sample was denoted as GTPC-0. The yields of GTPC-0, GTPC-1, GTPC-2, and GTPC-KOH were around 75, 60, 55, and 40 wt%, respectively.

### 2.3. Material Characterization

The surface morphologies of the samples were observed via ultra-high-resolution field-emission scanning electron microscopy (JEOL JSM-7610F Plus operated at 5 kV) and transmission electron microscopy (TEM; JEOL, JEM-2100 plus at 200 kV). In addition, X-ray diffraction (XRD) patterns were obtained with a Rigaku MiniFlex II XRD instrument with Cu-K_α_ radiation. The Raman spectra were collected using a Jobin Yvon (iHR-550) micro-Raman spectrometer under 488 nm laser illumination. The nitrogen adsorption–desorption isotherms were measured using an ASAP 2060 (Micromeritics) apparatus. The surface area (S), pore size distribution, pore volume (V), and micropore area were calculated using Brunauer–Emmett–Teller (BET) analysis, the Barrett–Joyner–Halenda (BJH) method, and the t-plot method. In addition, X-ray photoelectron spectroscopy (XPS; PHI-5000 Versa-Probe) was performed at room temperature using monochromatic Al Kα radiation.

### 2.4. Electrochemical Measurements

The electrochemical measurements were performed at an electrochemical workstation (SP-50/150, BioLogic). To test the applicability of the GTPC samples in supercapacitors, three-electrode measurements were performed. A Hg/HgO electrode and platinum plate counter electrode (1 × 1 cm) were used as the reference and counter electrodes, respectively. The working electrodes were prepared by mixing 80 wt% of the active material with 10 wt% super P and 10 wt% polyvinylidene fluoride (PVDF). A Ni foam substrate was used as the current collector. The loading mass was controlled within the range of 2 to 2.5 cm^2^. The gravimetric capacitance was calculated as follows:

(1) $$C_{g}=\frac{It}{m\Delta V}$$

where *C_g_* is the gravimetric capacitance (F g^−1^), *V* is a potential window, *I* is the current (A), *t* is the discharge time (s), and *m* is the mass of active materials (g).

The carbon cloth was cut into pieces (1 × 5 cm in area, 330 μm in thickness), and acted as the current collector. The working electrode materials were prepared by mixing 80 wt% of the active material with 10 wt% super P and 10 wt% PVDF. Thereafter, the electrode materials were pasted onto the carbon cloth within an area of 1 × 1 cm. The PVA/H_2_SO_4_ gel electrolyte was prepared as follows: 3 g of PVA was added to a 30 mL solution of 1 M H_2_SO_4_. The mixture was heated at 85 °C with stirring until it became transparent. It was then poured onto the two carbon electrodes. The electrodes, with a thin layer of gel electrolyte, were left in a fume hood at room temperature overnight to vaporize the solvent. Finally, the two pieces of electrode were assembled face-to-face under a pressure of 0.2 MPa for 10 min. The areal capacitance was calculated as follows:

(2) $$C_{a}=\frac{It}{A\Delta V}$$

Here, *C_a_* is the areal capacitance (mF cm^−2^), and *A* (cm^2^) is the projected area of the carbon cloth.

Furthermore, the areal energy density (*E_a_*, µWh cm^−2^) and area power density (*P_a_*, µW cm^−2^) of the devices were calculated as follows [19]:

(3) $$E_{a}=\frac{C_{a}\Delta V^{2}}{2\times 3.6}$$

(4) $$P_{a}=\frac{3600E_{a}}{t}$$

## 3. Results and Discussion

**Scheme 1:**
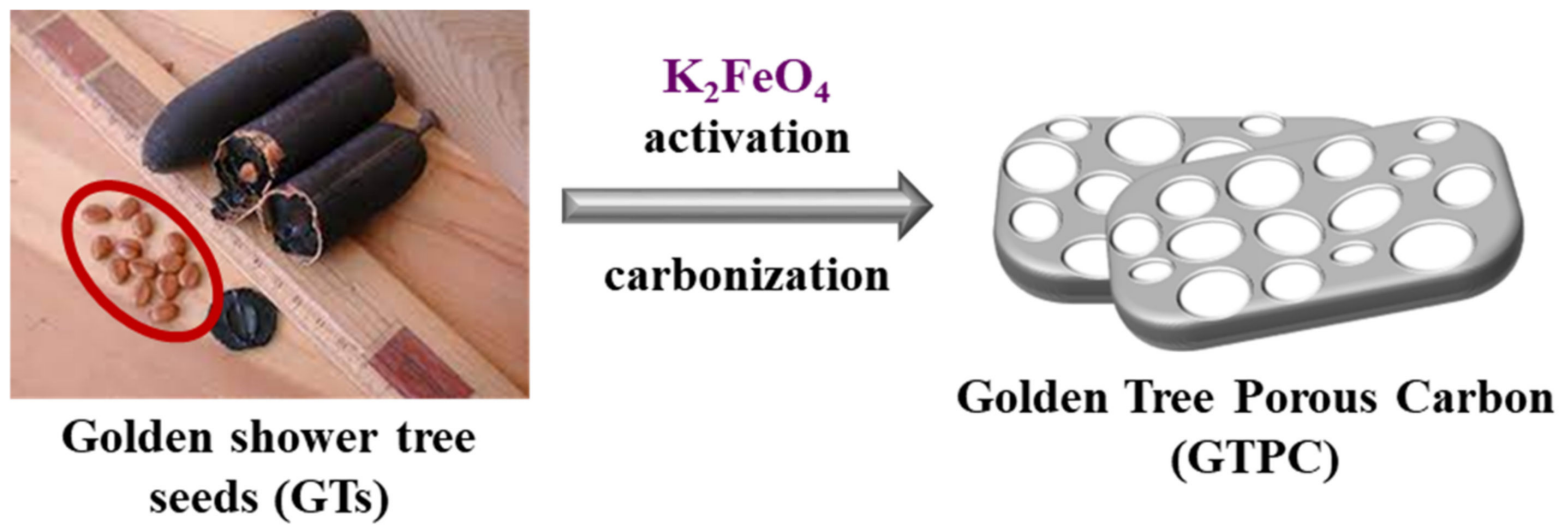
Schematic illustration of synthesis of golden shower tree derived porous carbon (GTPC), with golden shower tree seeds (GTs) and K_2_FeO_4_ as the activation agent.

Golden shower trees are mostly found in tropical and subtropical regions. The fabrication procedure for GTPC, using GTs as carbon sources and K_2_FeO_4_ as the activation reagent, is schematically illustrated in Scheme 1. The GTs were ground into a powder using a milling machine, and were then mixed with the K_2_FeO_4_. The mixed samples were further carbonized at 800 °C for 2 h to convert them into activated porous carbon. The samples were denoted as GTPC-0, GTPC-1, and GTPC-2, according to the weight ratio as described in the Experimental Section. After removing residual metal species with an 8% HF solution, we obtained GTPC samples with highly porous structures. The comparison sample using KOH as the activating agent was fabricated using the same fabrication process, and was denoted as GTPC-KOH.

**Figure 1:**
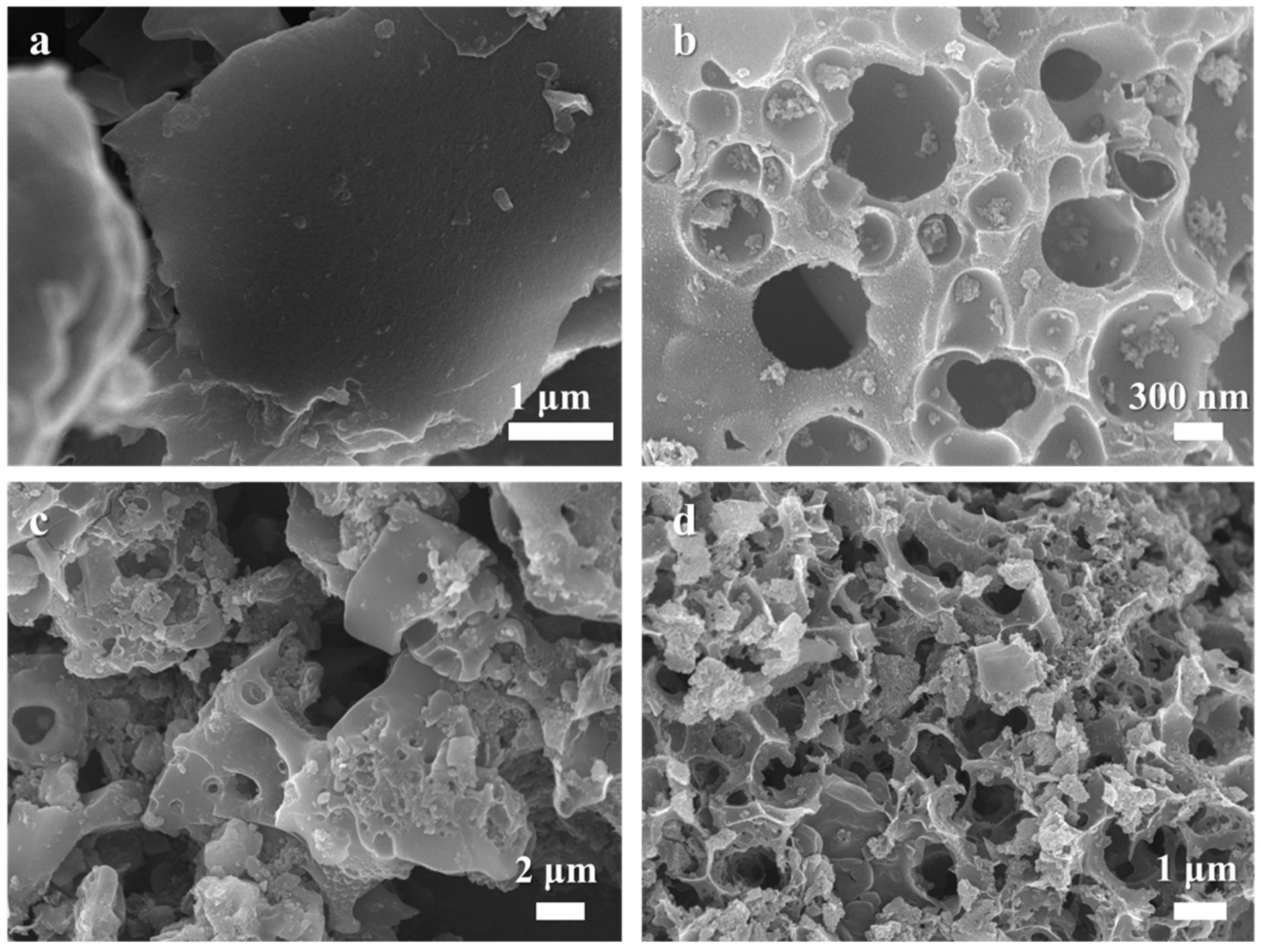
Scanning electron microscope (SEM) images of (**a**) GTPC-0, (**b**) GTPC-1, (**c**) GTPC-2, and (**d**) GTPC-KOH.

**Figure 2:**
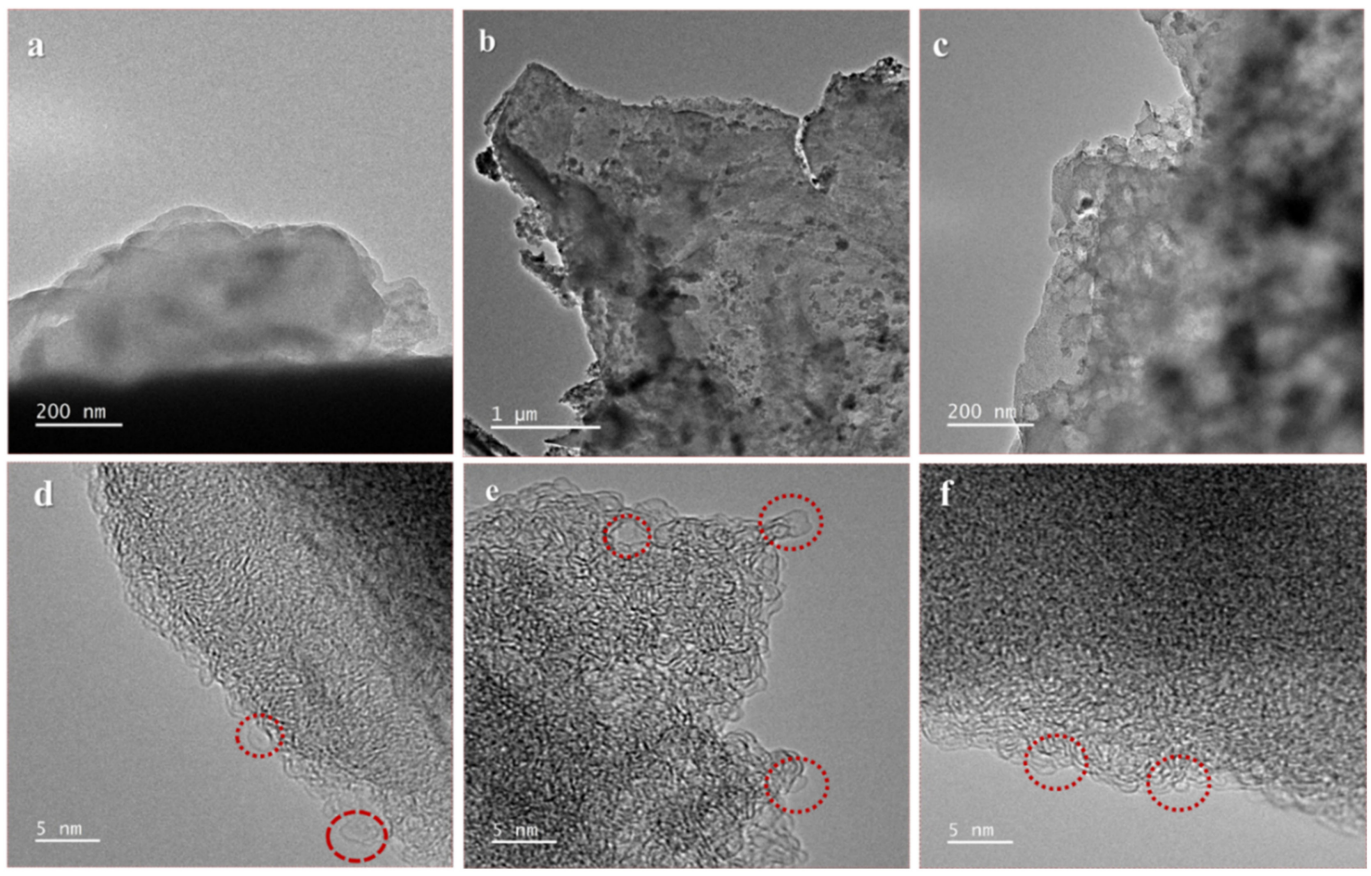
TEM and HR-TEM images of (**a**,**d**) GTPC-0, (**b**,**e**) GTPC-1, and (**c**,**f**) GTPC-KOH.

The morphologies and microstructures were characterized by scanning electron microscopy and transmission electron microscopy (TEM) (Figure 1 and Figure 2). GTPC-0, which was directly carbonized from GTs without any activation treatment, exhibited a smooth and flat surface (Figure 1a). In Figure 1b–d, the GTPC samples show holey morphologies and possesses apparent porous structures. GTPC-1 and GTPC-2 were etched with K_2_FeO_4_, whereas GTPC-KOH was activated by KOH. At high temperatures, utilizing K_2_FeO_4_ as a simultaneous activation and graphitization agent can enable a dissolution–precipitation reaction of the graphitized carbon, which might promote the formation of micropores and mesopores [16,20,21]. The porous structure was characterized using high-resolution TEM. As shown in Figure 2, GTPC-1 contains distinct pores, as indicated by the red dashed lines; in contrast, few pores can be observed in the other GTPC samples.

**Figure 3:**
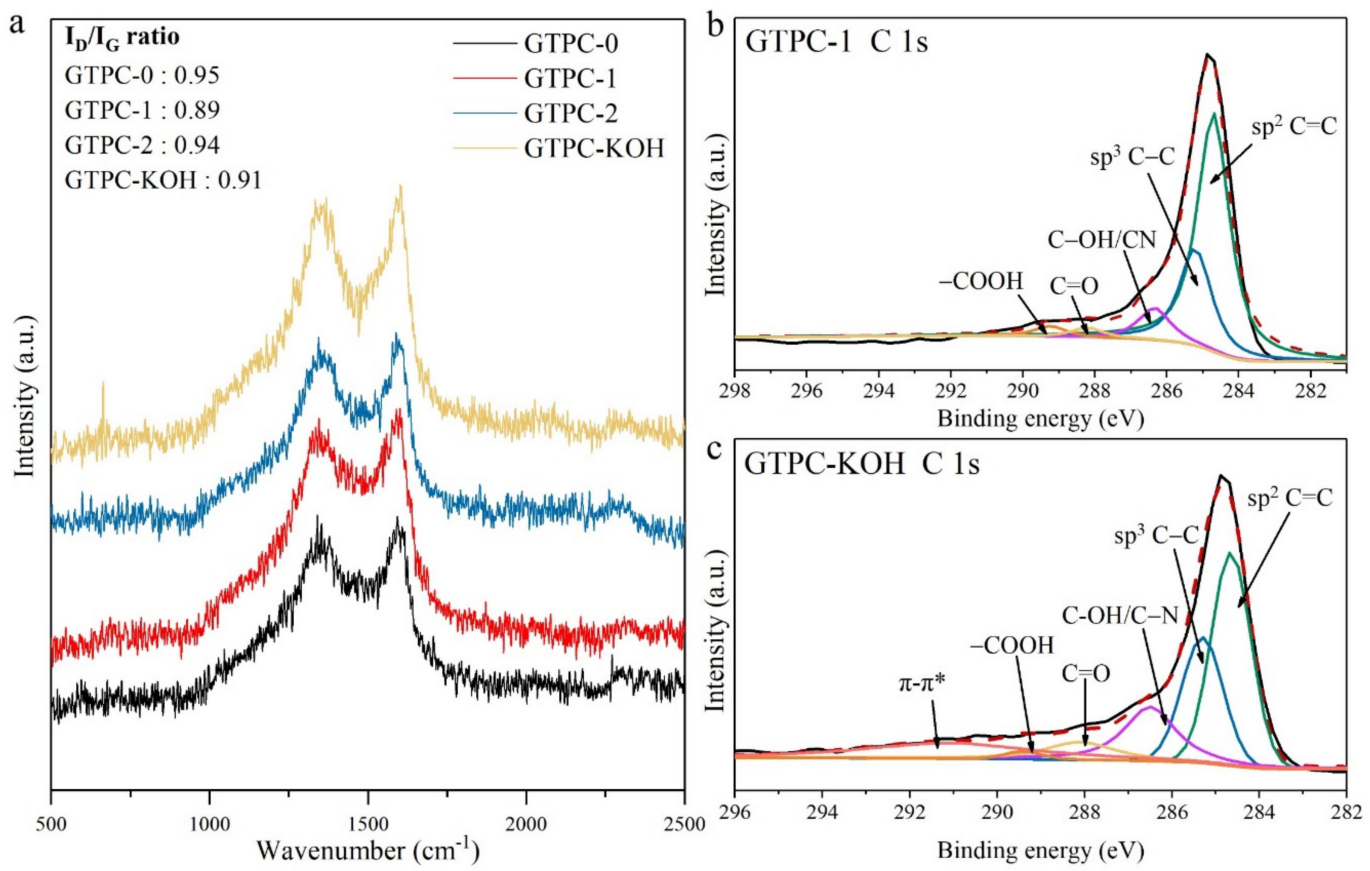
(**a**) Raman spectroscopy of GTPC samples and C 1s X-ray photoelectron spectroscopy (XPS) spectra of (**b**) GTPC-1 and (**c**) GTPC-KOH.

The XRD patterns of the GTPC samples are shown in Figure S1. The wide-angle XRD patterns showed two evident diffraction peaks at 25° and 44°, corresponding to the (002) and (110) planes of the carbon, respectively [22]. The presence of these broad peaks suggested the formation of amorphous carbon in all of the GTPC samples. The graphitized structures were further analyzed by Raman spectroscopy (Figure 3a). The prominent peaks at 1338 and 1590 cm^−1^ were assigned to the disordered (D) and graphitic (G) peaks of the carbon, respectively. The D-band arising from the sp^3^-bonded carbon structure or defects is a characteristic of many defect-containing carbons, among which the presence of mainly amorphous carbon is the most important contribution. The G-band corresponds to the sp^2^-bonded carbon in the graphitic layers. The ratio of the D-band intensity to the G-band intensity (I_D_/I_G_) can be used to characterize amorphous or defect-containing carbon materials. A higher I_D_/I_G_ value indicates a higher disorder in the material, and a lower degree of graphitization. The I_D_/I_G_ values for GTPC-0, GTPC-1, GTPC-2, and GTPC-KOH were 0.95, 0.89, 0.94, and 0.91, respectively. The results indicated that the ordering and graphitization degrees of the activated carbons (GTPC-1, GTPC-2, and GTPC-KOH) were slightly higher than those of GTPC-0. GTPC-1 presented the lowest I_D_/I_G_ value, suggesting the successful formation of graphitic carbon owing to the catalytic effect of the Fe species. An X-ray photoelectron spectroscopy (XPS) analysis was performed to investigate the details of the contents and band structures of the carbon in all of the samples (Figure 3b,c and Figure S2). The C 1s spectrum can be resolved into six distinct peaks, corresponding to C=C, C−C, C−OH/CN, C=O, COOH, and π−π* [23]. The ratio of C=C (sp^2^) to C−C (sp^3^) was the highest for GTPC-1 (Figure 2b); this agreed with the Raman results. The O1s (Figure S2c,e,g) spectra exhibited three fitted peaks; these could be attributed to the components of C=O, C−O, and O−H. The N 1s spectrum (Figure S3d,f,h) can be divided into four peaks, corresponding to pyridinic N, pyrrolic N, quaternary N, and graphitic N. The presence of heteroatoms and functional groups in carbon provide an effective way to modify the intrinsic physicochemical properties of the carbon [24]. In particular, N-doped carbon materials can cause increases in capacitance and electrical conductivity, owing to the electron/donor effect between the nitrogen and carbon [25]. The XPS results indicated that N species were present in the carbon frameworks. The elemental compositions of all of the samples are shown in Table S1. GTPC-1 and GTPC-2 possessed higher nitrogen than the other two samples, indicating the advantages of reserving nitrogen components during the carbonization and K_2_FeO_4_ activation treatment. In addition, GTPC-1 had the highest carbon composition, showing the best conversion of carbon. Due to the activation process with higher concentration of K_2_FeO_4_, GTPC-2 presented lower carbon and higher oxygen compositions than GTPC-1. Compared with K_2_FeO_4_, the etching effect of KOH was higher, causing the lowest carbon composition and the highest oxygen composition in GTPC-KOH.

**Figure 4:**
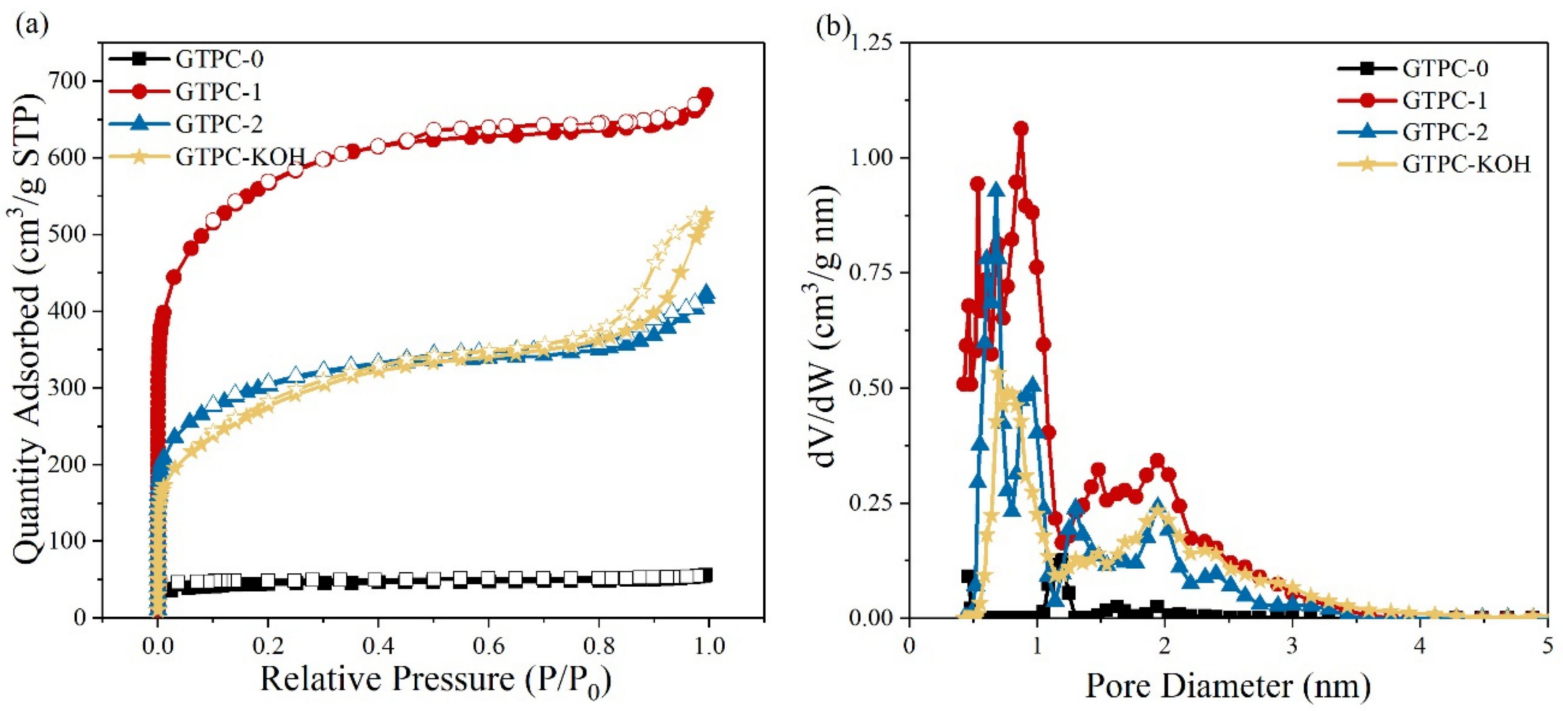
(**a**) Nitrogen adsorption–desorption isotherm and (**b**) pore size distribution of GTPC samples.

The surface area and pore size distribution are key factors determining the electrochemical performance of electrode materials. Accordingly, nitrogen adsorption–desorption measurements were performed to investigate the porous structures of the GTPC samples. As shown in Figure 4a, the isotherms of the GTPC-1, GTPC-2, and GTPC-KOH samples were classified as type I and IV curves, where sharp uptake slopes are present in the low-pressure range and hysteresis loops appear in the relative pressure range of 0.3 to 0.95, suggesting their microporous and mesoporous nature [26]. The specific surface areas, pore volumes, micropore areas, and percentages of micropores are listed in Table 1. The specific surface areas of GTPC-0, GTPC-1, GTPC-2, and GTPC-KOH as calculated using the BET method were 115, 1915, 1029, and 978 m^2^ g^−1^, respectively. The pore size distribution (Figure 4b) and pore volume calculations were based on the BJH calculations. The percentages of the micropores (S_micro_/S_BET_) were calculated using the t-plot method. Compared with GTPC-0, the other three samples, with the aid of an activation agent, had significantly higher specific surface areas and pore volumes. This suggested that heat treatment with chemical activation successfully carbonized the GTs into highly porous carbon. Larger pores provide more active sites for ions to transfer within the electrolyte and electrode surface, and enhance the rate of electrochemical reactions [27]. Among all of the samples, the GTPC-1 sample had the highest specific surface area (1915 m^2^ g^−1^) and pore volume (1.045 cm^3^ g^−1^), high micropore area (795 m^2^ g^−1^), and a higher presence of mesopores. Although activating with higher concentration of K_2_FeO_4_, the GTPC-2 sample presented a significantly lower specific surface area than GTPC-1, indicating that the overetching may affect the porous structure. These results show that the chemical activation of K_2_FeO_4_ with a weight ratio of 1:1 is an effective way to create abundant pores.

**Figure 5:**
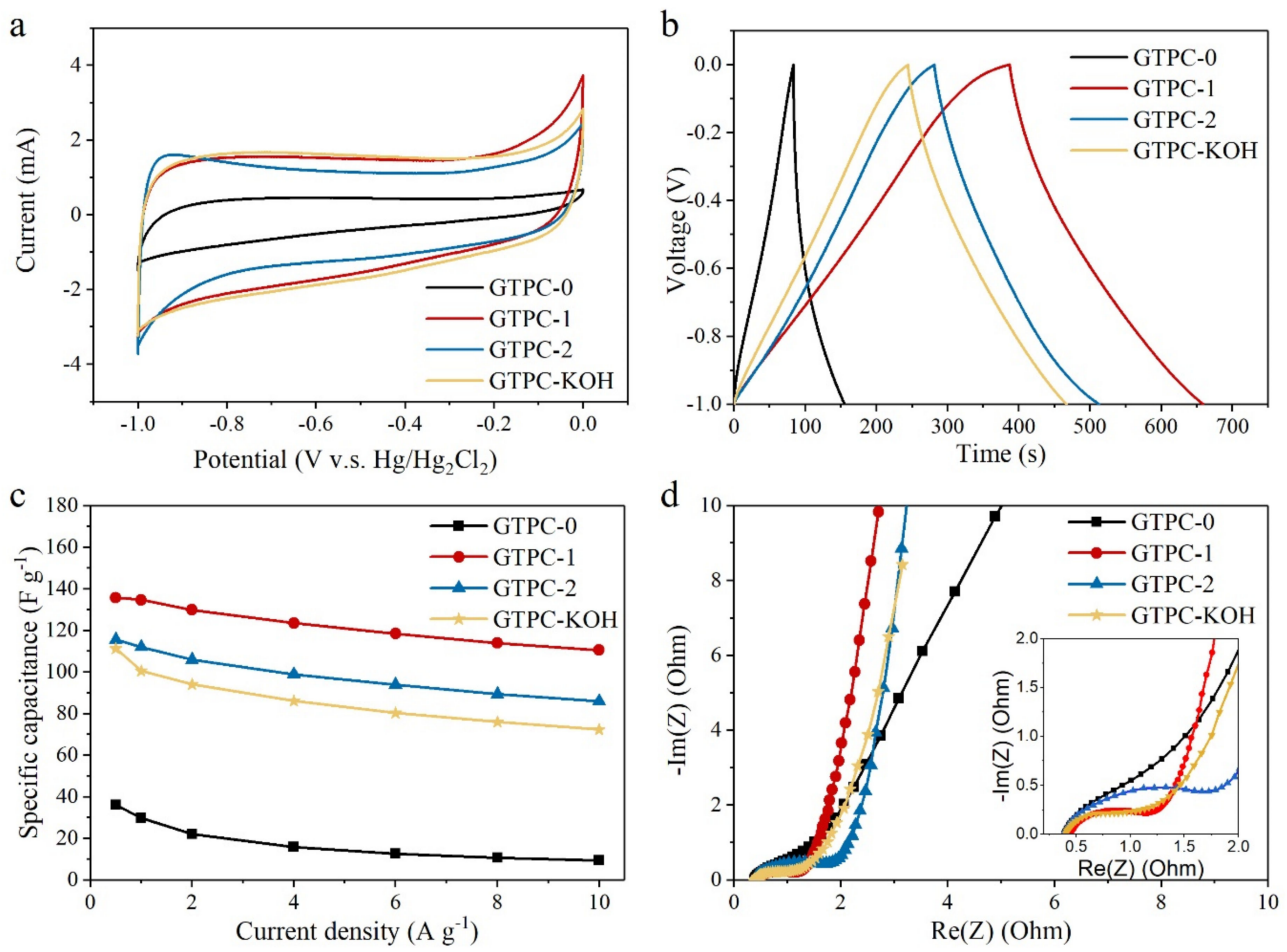
(**a**) Cyclic voltammetry (CV) curves of all of the GTPC samples at the scan rate of 5 mV s^−1^. (**b**) Charge–discharge curves of GTPC samples at the current density of 0.5 A g^−1^. (**c**) Specific capacitances of GTPC samples at different current densities. (**d**) Nyquist plots of all of the GTPC samples.

The electrochemical performance of all of the GTPC samples was analyzed using a three-electrode system in a 6 M KOH electrolyte. The cyclic voltammetry (CV) measurements are shown in Figure 5a. All curves displayed a well-developed rectangular shape, revealing typical electric double-layer capacitor (EDLC) characteristics. The optimal potential window was adjusted to −1 to 0 V. In addition, galvanostatic discharge/charge (GCD) measurements were conducted at different current densities. The specific capacitances of GTPC-0, GTPC-1, GTPC-2, and GTPC-KOH were 36.1, 135.8, 115.6, and 111.2 F g^−1^, respectively, at 0.5 A g^−1^ (Figure 5b). GTPC-0 showed the lowest specific capacitance at all current densities, owing to its lowest specific surface area and pore volume. GTPC-1 exhibited the highest specific capacitance among all of the samples, owing to its well-constructed porous structure, high surface area, and optimized micro–mesoporous structure. The rate capability, as determined by GCD measurements, is another crucial factor for high-performance supercapacitors. The specific capacitance versus current density (from 0.5–10 A g^−1^) curves of all samples are displayed in Figure 5c. The rate capabilities of all the samples were calculated as 26.26%, 82.06%, 74.42%, and 65.04%, respectively. GTPC-1 featured a high capacitance retention of 82.06% (~110.6 F g^−1^) at a high current density of 10 A g^−1^, which is highly suitable for EDLCs. This may be owing to its high carbon content (86.1.%), graphitized carbon structure (sp^2^ carbon), high specific surface area, large pore volume, and the presence of mesopores. As a result, the electrical conductivity increased. Electrochemical impedance spectroscopy (EIS) measurements were performed to investigate the resistance of all of the samples. As shown in Figure 5d, the Nyquist plots of all samples show a partial semicircle in the high-frequency region, and a straight line in the low-frequency region. GTPC-1 exhibits a steeper slope of the inclined line, indicating a relatively lower diffusion resistance between the electrolyte and electrode material. Moreover, the diameter of the partial semicircle of GTPC-1 is relatively smaller than GTPC-0 and GTPC-2, indicating a lower charge transfer resistance. This result is in good accordance with previous characterization and electrochemical tests, demonstrating that the improvement in the electrical conductivity and capacitance retention was related to the presence of the mesopores and sp^2^ carbon structure.

**Figure 6:**
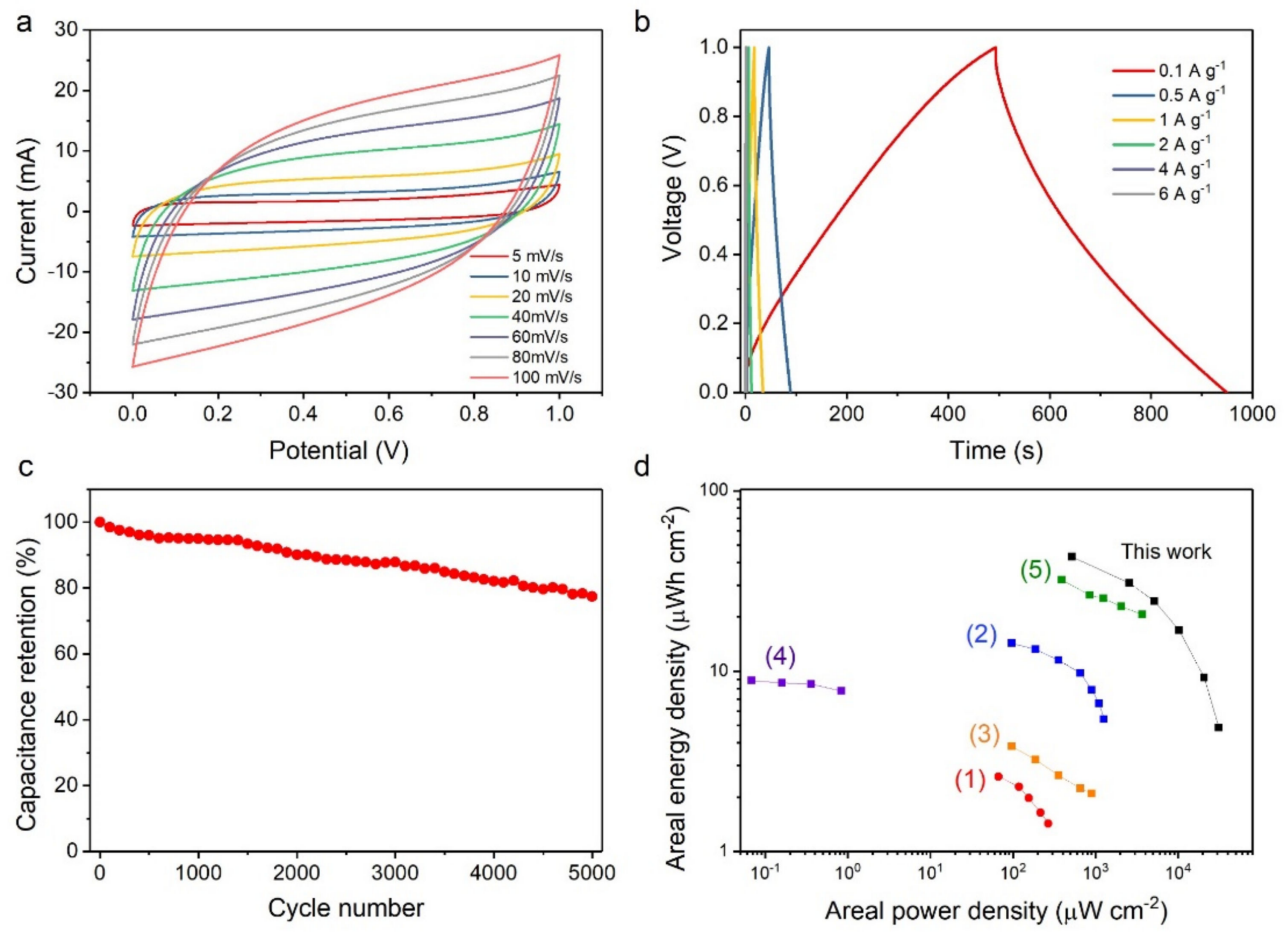
(**a**) CV curves of GTPC-1-based solid-state supercapacitor at various scan rates. (**b**) Discharge curves of GTPC-1-based solid-state supercapacitor at different current densities. (**c**) Cycling stability of the solid-state supercapacitor for 5000 cycles at a current density of 1 A g^−1^. (**d**) Comparison of our solid-state supercapacitor with previous reported solid-state supercapacitors from related literature ((1) Carbon nanotube/MnO_2_/polymer fiber [33], (2) polyaniline/polyvinyl alcohol (PANI–PVA) composite hydrogel [34], (3) reduced graphene oxide(RGO) + carbon nanotube(CNT)@ceramic matrix composites fibers [35], (4) Ag–Doped poly(3,4-ethylenedioxythiophene) polystyrene sulfonate/CNT composites [31], and (5) Mn_3_O_4_/RGO/single-walled CNT hybrid film-based solid-state supercapacitor. [28]).

To further explore the advantages of the GTPC-1 material in flexible and wearable electronic devices, a symmetric solid-state supercapacitor device was assembled, with two pieces of GTPC-1 on each side of a PVA/H_2_SO_4_ gel electrolyte (Figure S4). The assembled device showed flexibility (Figure S5). CV tests were performed at scan rates ranging from 5 to 100 mV s^−1^ (Figure 6a). The CV curves featured typical EDLC characteristics, illustrating good reversibility and a fast current response. Figure 6b shows the discharge curves of the device at various current densities, ranging from 0.1 to 6 A g^−1^. Electrochemical cycling tests were conducted for 5000 cycles at a current density of 1 A g^−1^ (Figure 6c). As shown in Figure S6, the Nyquist plot shows a semicircle and a nearly vertical line in the high-and low-frequency ranges, respectively. The solid-state supercapacitor exhibits a very low equivalent series resistance of ~3.1 Ω. A comparison of our device to those from other studies is listed in Table S2. Our device shows an excellent specific areal capacitance and comparable capacitance retention relative to the other devices reported in the literature [28,29,30,31,32].

To evaluate the performance of the solid-state supercapacitor device, the areal energy density and areal power density were calculated and plotted on a Ragone plot (Figure 6d). A direct comparison of our device with others showed that our device had similar or better performance [28,31,33,34,35]. It provided a high areal energy density of 42.93 µWh cm^−2^, corresponding to an areal power density of 520 µW cm^−2^ at a current density of 0.1 A g^−1^. At 6 A g^−1^, the areal power density was 31.2 mW cm^−2^, corresponding to an areal energy density of 4.85 µWh cm^−2^.

With the depletion of energy and deteriorating environment arising from recent overdependence on fossil fuels, it is important to develop sustainable technologies. Biomass-derived carbons are considered the next generation of electrode materials for energy storage devices, owing to their abundance and environmental friendliness. The design of biomass-derived carbon materials with appropriate structural and surface chemistry properties is important for their applications in energy storage devices. In this regard, our GTPC samples are potential materials for electrodes and exhibit outstanding electrochemical performance owing to their several advantages, e.g., highly porous structure, high surface area, optimized micro–mesoporous structure, high carbon content, and graphitized structure. Moreover, our facile approach to assembling a solid-state supercapacitor based on the biomass-derived carbon provides an economic method for fabricating thin films and bendable supercapacitors for use in wearable electronics in the future.

## 4. Conclusions

A facile and scalable approach for fabricating activated porous carbon from GTs was developed for supercapacitor applications. We confirmed that the activating agent and concentration are both crucial for fabricating highly porous carbon. The obtained carbon, GTPC-1, possessed a high specific surface area and contained graphitic carbon, benefiting from the catalytic effect of the K_2_FeO_4_. When evaluated as electrodes for supercapacitors, GTPC-1 showed an excellent electrochemical performance. Therefore, a solid-state supercapacitor based on GTPC-1 was assembled, which exhibited a high areal energy density and areal power density with good cycling performance. The superior electrochemical performance is ascribed to the combined merits of the well-constructed porous structure, high surface area, and optimized microporous structure. We think that these insights into transforming biomass materials into highly porous activated carbon will be of significance in the green production of potential electrode materials for high-performance supercapacitors and energy storage devices.

## Figures and Tables

**Table 1:** Specific surface areas, pore volume, micropore areas, and percentages of micropores of GTPC samples.

Sample	Specific Surface Area (S_BET_, m^2^ g^−1^)	Pore Volume (cm^3^ g^−1^)	Micropore Area (S_micro_, m^2^ g^−1^)	S_micro_/S_BET_ (%)
GTPC-0	115	0.061	77	67.0
GTPC-1	1915	1.045	795	41.5
GTPC-2	1029	0.632	413	40.1
GTPC-KOH	978	0.799	134	13.7

## Supplementary Materials

The following are available online at https://www.mdpi.com/article/10.3390/mi13060916/s1, References [28,29,30,31,32,36] are cited in Supplementary. Figure S1: XRD patterns of GTPC-0, GTPC-1, GTPC-2, and GTPC-KOH, Figure S2: C 1s, O 1s and N 1s spectra of GTPC-0, GTPC-1, GTPC-2, and GTPC-KOH, Figure S3: Cyclic voltammetry (CV) curves and Charge-discharge curves of GTPC-0, GTPC-1, GTPC-2, and GTPC-KOH samples, Figure S4: The photograph and schematic illustration of solid-state flexible GTPC-1 supercapacitor based on PVA/H2SO4 polymer gel electrolyte, Figure S5: The photograph of solid-state flexible supercapacitor device, Figure S6: Nyquist plot of the solid-state supercapacitor based on GTPC-1, Table S1: The proportion of carbon, oxide and nitrogen calculated by XPS, Table S2. Comparison of electrochemical performance of solid-state supercapacitors reported in previous literatures.
