# Supplementary material for: Highly Porous Holey Carbon for High Areal Energy Density Solid-State Supercapacitor Application
Source: Micromachines (Basel). 2022 Jun 9;13(6):916. doi: 10.3390/mi13060916 (PMC9229398; doi:10.3390/mi13060916)
Supplement: Supplementary file 1 [file micromachines-13-00916-s001.zip › micromachines-1743715-supplementary.pdf]

Supplementary

Highly Porous Holey Carbon for High Areal Energy Density Solid-State Supercapacitor Application

Table S1. The proportion of carbon, oxide and nitrogen calculated by XPS.

Sample	C (at.%)	O (at.%)	N (at.%)
**GTPC-0**	74.4	23.7	1.8
**GTPC-1**	86.1	10.6	3.3
**GTPC-2**	78.5	14.8	6.6
**GTPC-KOH**	63.5	35.2	1.3

Table S2. Comparison of electrochemical performance of solid-state supercapacitors reported in previous literatures.

Electrode materials	Specific capacitance for electrode	Areal capacitance for supercapacitor device	Electrolyte	Capacitance retention	References
GTPC-1	135.8 F g^{-1} (0.5 A g^{-1})	361.3 mF cm^{-2} (5 mV s^{-1})	PVA/H_2SO_4	90.1% (1 A g^{-1}, 1000 cycles)	This work
Mn_3O_4/RGO/SWCNT	796 mF cm^{-2}(1 mA cm^{-2})	360 mF cm^{-2} (1 mA cm^{-2})	PVA/KOH	95% (5000 cycles)	[28]
BP/MnO2//CFT/GPs	-	163.5 mF cm^{-2} (0.4 mA cm^{-1})	PVA/KOH	95% (3 mA cm^{-1}, 5000 cycles)	[29]
PANI	-	11.3 mF cm^{-2} (5mV s^{-1})	PVA/H_2SO_4	83% (0.1 mA cm^{-2}, 1000 cycles)	[30]
Ag–doped PEDOT:PSS/CNT	-	64 mF cm^{-2}	PVA/H_3PO_4	-	[31]
CNT/MnO_2	-	73 mF cm^{-2} (0.6 mA cm^{-2})	PVA/KOH	97.8% (1 V s^{-1}, 20000 cycles)	[32]

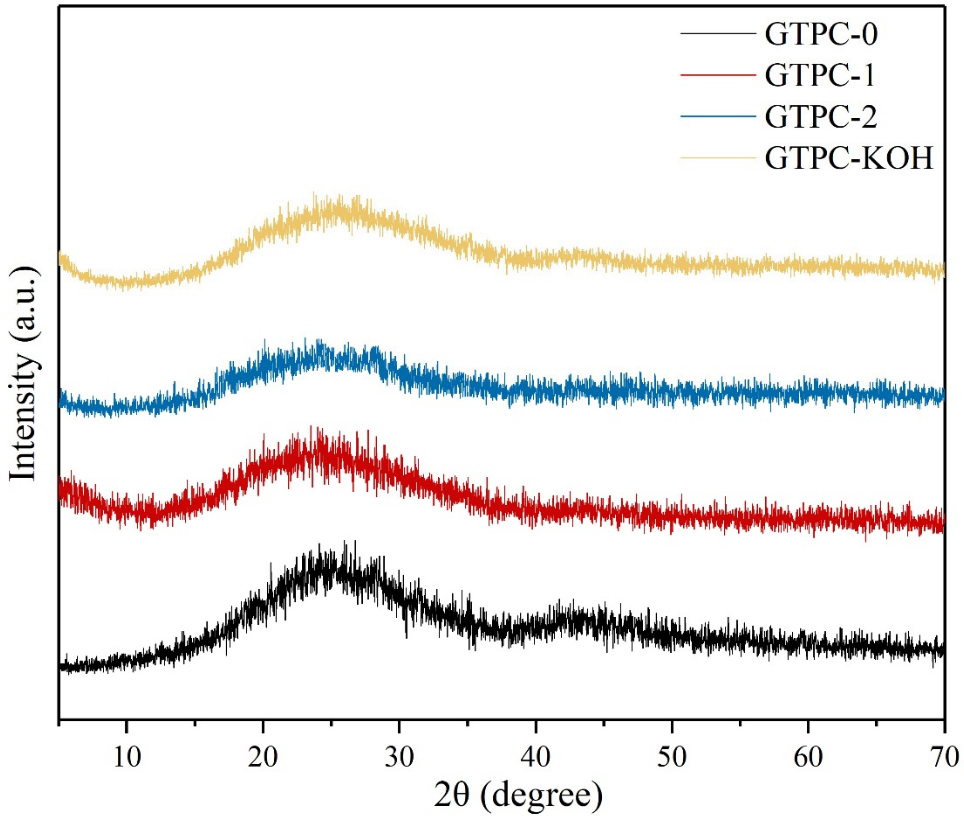

Figure S1. XRD patterns of GTPC-0, GTPC-1, GTPC-2, and GTPC-KOH.

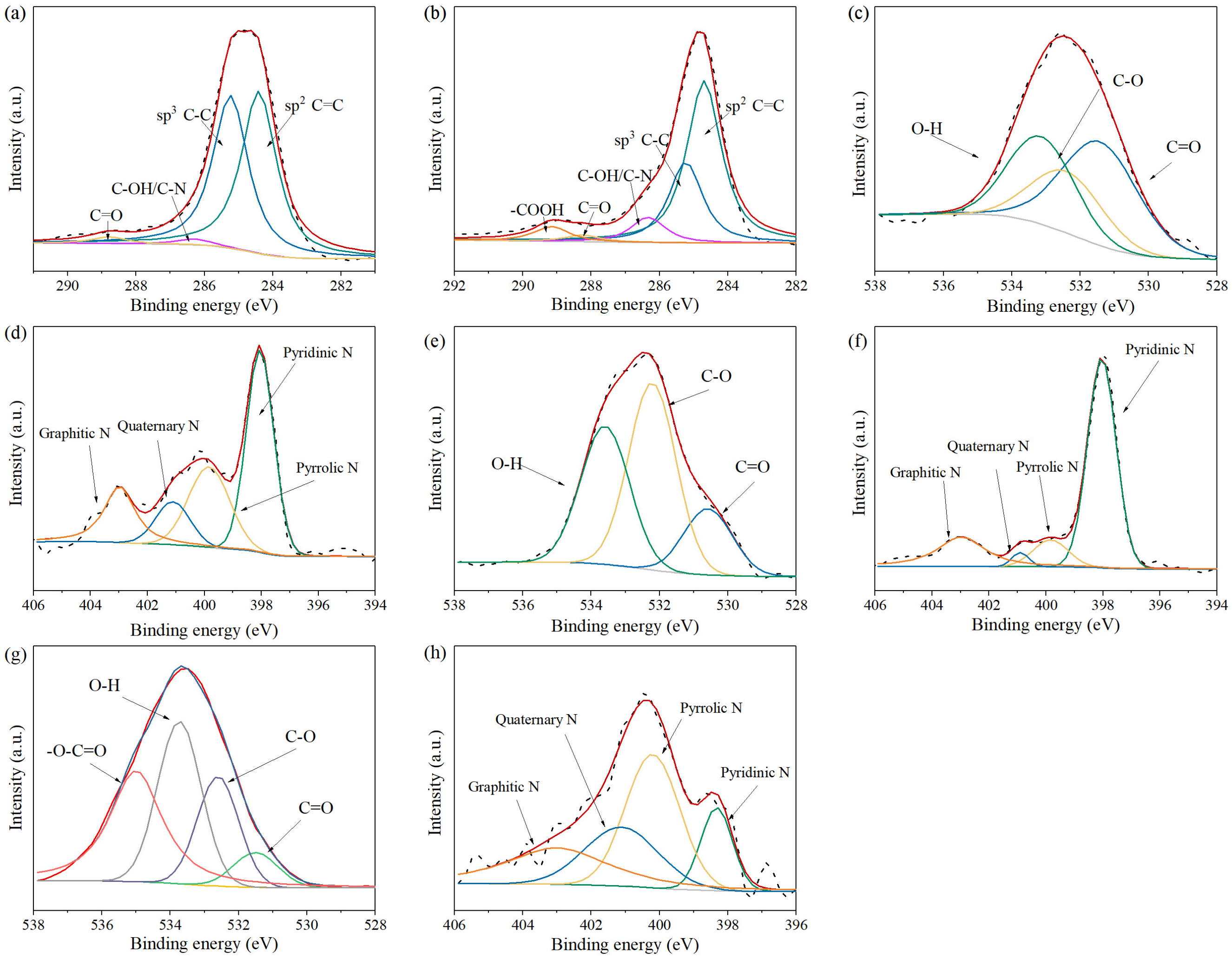

Figure S2. C 1s spectra of (**a**,**b**) GTPC-0 and GTPC-2. (**c**,**e**,**g**) O 1s spectra of GTPC-1, GTPC-2, GTPC-KOH. (**d**,**f**,**h**) N 1s spectra of GTPC-1, GTPC-2, GTPC-KOH.

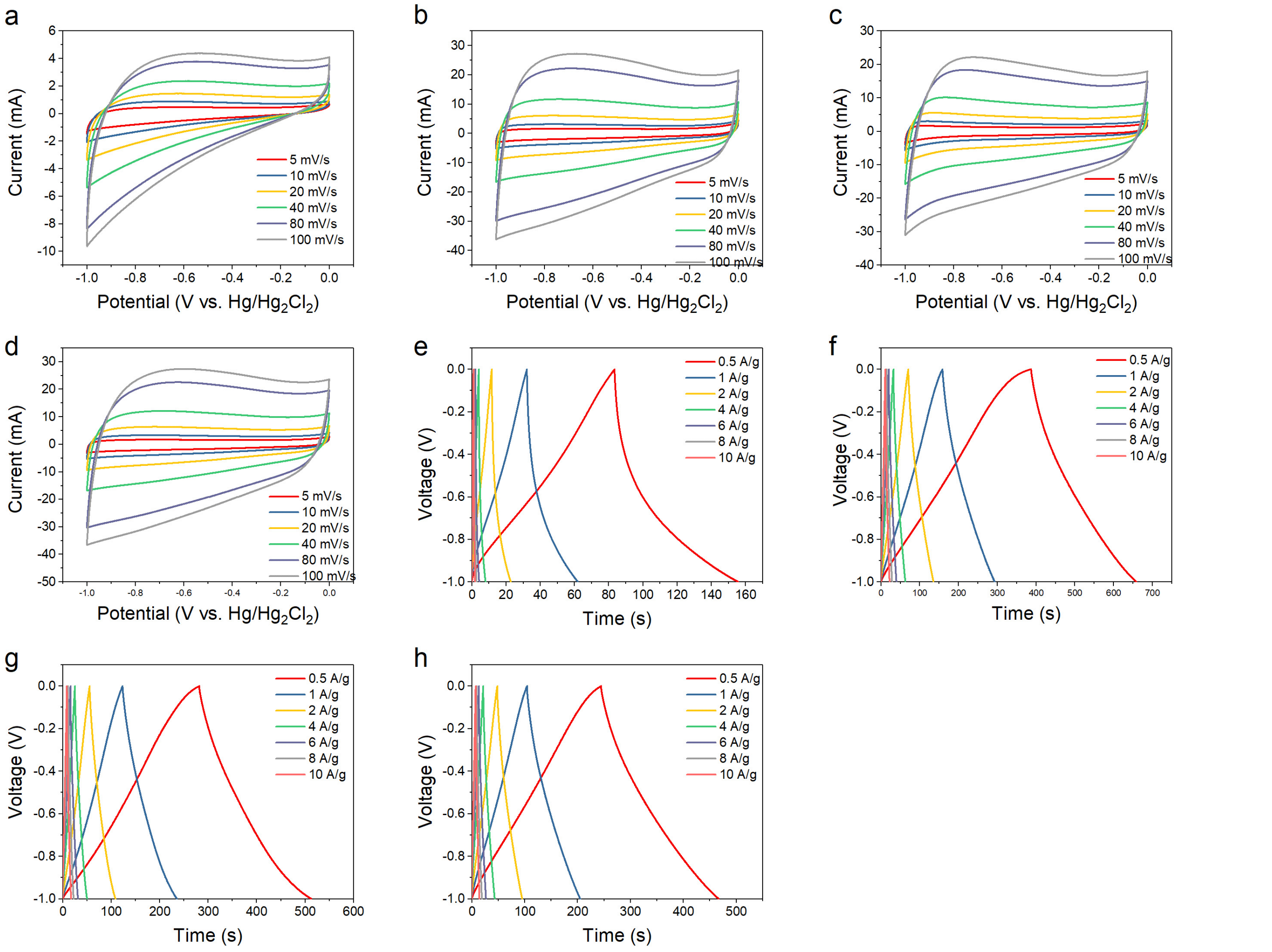

Figure S3. (**a–d**) Cyclic voltammetry (CV) curves of GTPC-0, GTPC-1, GTPC-2, and GTPC-KOH samples at the scan rate of 5 to 100 mVs^{-1}. (**e–h**) Charge-discharge curves of GTPC-0, GTPC-1, GTPC-2, and GTPC-KOH samples at the current density of 0.5 to 10 A g^{-1}.

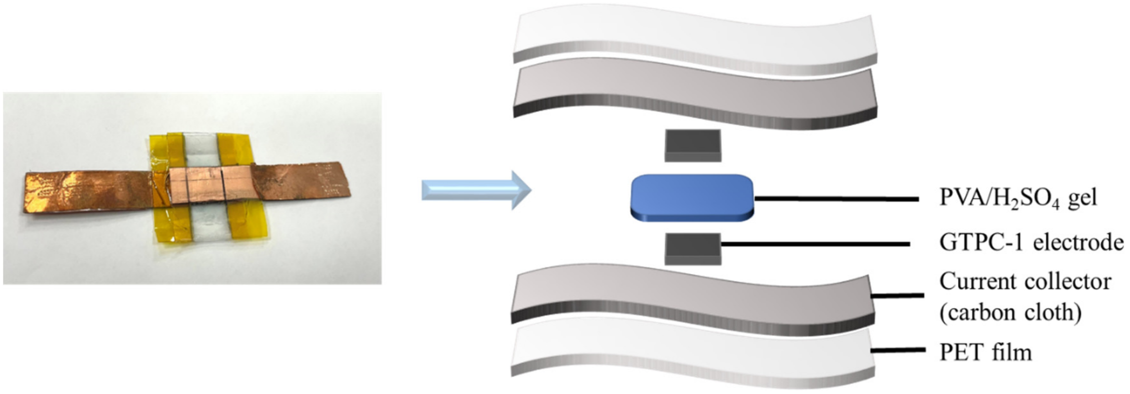

Figure S4. The photograph and schematic illustration of solid-state flexible GTPC-1 supercapacitor based on PVA/H_2SO_4 polymer gel electrolyte.

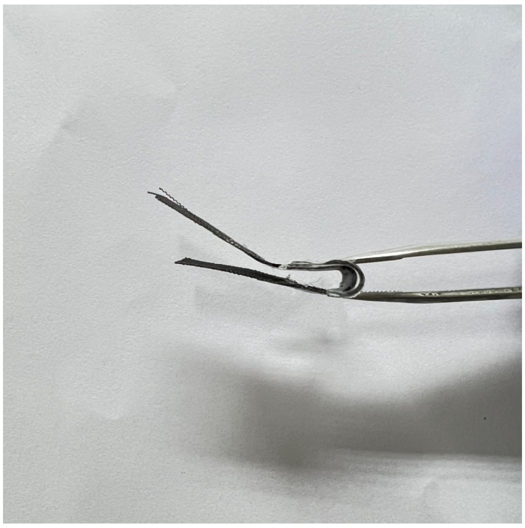

Figure S5. The photograph of solid-state flexible supercapacitor device.

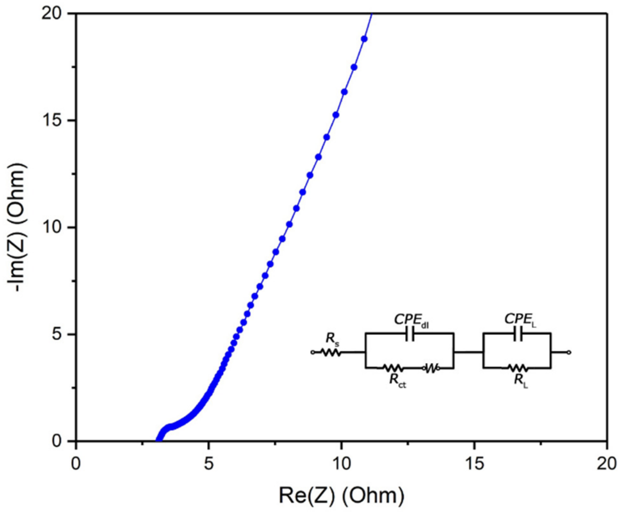

Figure S6. Nyquist plot of the solid-state supercapacitor based on GTPC-1. The inset shows the equivalent circuit of Randle's model, where R_s is is the equivalent series resistance, R_{ct} is the charge transfer resistance of the electrode-electrolyte, CPE_{dl} is the constant phase element of double layer, W is the Warburg element, CPE_L is the mass capacitance [36].
